# Prediction of VO2max From Submaximal Exercise Using the Smartphone Application Myworkout GO: Validation Study of a Digital Health Method

**DOI:** 10.2196/38570

**Published:** 2022-08-04

**Authors:** Jan Helgerud, Håvard Haglo, Jan Hoff

**Affiliations:** 1 Department of Circulation and Medical Imaging Faculty of Medicine and Health Sciences Norwegian University of Science and Technology Trondheim Norway; 2 Medical Rehabilitation Clinic Myworkout Trondheim Norway; 3 Faculty of Health and Social Sciences Molde University College Trondheim Norway

**Keywords:** high-intensity interval training, cardiovascular health, physical inactivity, endurance training, measurement accuracy

## Abstract

**Background:**

Physical inactivity remains the largest risk factor for the development of cardiovascular disease worldwide. Wearable devices have become a popular method of measuring activity-based outcomes and facilitating behavior change to increase cardiorespiratory fitness (CRF) or maximal oxygen consumption (VO_2max_) and reduce weight. However, it is critical to determine their accuracy in measuring these variables.

**Objective:**

This study aimed to determine the accuracy of using a smartphone and the application Myworkout GO for submaximal prediction of VO_2max_.

**Methods:**

Participants included 162 healthy volunteers: 58 women and 104 men (17-73 years old). The study consisted of 3 experimental tests randomized to 3 separate days. One-day VO_2max_ was assessed with Metamax II, with the participant walking or running on the treadmill. On the 2 other days, the application Myworkout GO used standardized high aerobic intensity interval training (HIIT) on the treadmill to predict VO_2max_.

**Results:**

There were no significant differences between directly measured VO_2max_ (mean 49, SD 14 mL/kg/min) compared with the VO_2max_ predicted by Myworkout GO (mean 50, SD 14 mL/kg/min). The direct and predicted VO_2max_ values were highly correlated, with an R^2^ of 0.97 (*P*<.001) and standard error of the estimate (SEE) of 2.2 mL/kg/min, with no sex differences.

**Conclusions:**

Myworkout GO accurately calculated VO_2max_, with an SEE of 4.5% in the total group. The submaximal HIIT session (4 x 4 minutes) incorporated in the application was tolerated well by the participants. We present health care providers and their patients with a more accurate and practical version of health risk estimation. This might increase physical activity and improve exercise habits in the general population.

## Introduction

Physical inactivity is one of the leading health problems in the world. Exercise is important for rehabilitation, to enhance health, and for health maintenance, in addition to its role in conditioning for competitive sports [1-3]. Robust evidence shows that low levels of cardiorespiratory fitness (CRF) are associated with a high risk of cardiovascular disease and all-cause mortality. CRF, typically assessed by directly measuring maximal oxygen consumption (VO_2max_), is a potentially stronger predictor of mortality than established risk factors such as smoking [4]. The addition of CRF to traditional risk factors could lead to improved clinical practice and public health.

Indirect estimates of CRF have been associated with health outcomes for more than 50 years. There is a high correlation between cardiac output during exercise and VO_2_ [5]. A low heart rate (HR) at a given VO_2_ is thus associated with a large stroke volume. This physiological fact forms an important basis for submaximal exercise tests. Most modern circulatory exercise tests are based on the linear increase in HR with increasing VO_2_. However, only a few studies have established these prediction equations [4,6].

CRF has usually been estimated using maximal treadmill and bike testing [7-9]. However, a submaximal exercise test can be chosen when the apparatus and trained personnel to perform direct VO_2max_ measurements are either not available or considered inappropriate [5]. In addition, many researchers and clinicians are not willing to accept the definite risk involved in an incremental test to exhaustion. Submaximal exercise tests based on the HR response to work rate can be performed with little risk to the participant. However, the usefulness of CRF prediction must be considered with regard to the relatively large standard error of the estimate (SEE), which is typically in the range of more than 10% to 15% [4,6].

Wearable devices have become a popular method in health care and clinical research for measuring both activity-based outcomes and CRF. In a randomized controlled trial with patients with an inflammatory rheumatic disease, we recently documented the effect of a smartphone-assisted high aerobic intensity interval training (HIIT) with the app Myworkout GO [10]. Similar improvements in VO_2max_ and health-related quality of life were observed following HIIT when patients with an inflammatory rheumatic disease were guided by health care professionals or the training was self-administred and app-guided with CRF exercise feedback. Digital rehabilitation appears to be an excellent, cost-effective treatment strategy and should be considered in clinical practice in the future. It is thus critical to understand the accuracy when measuring theses variables because it may affect research conclusions and impact health care decision-making. Since wearable technology companies are solely responsible for reporting the accuracy of their products, little information about the evaluation method is made publicly available [11-13].

Although a number of risk scores combining multiple variables have been developed and validated as prognostic tools, we sought to predict VO_2max_ and thus “biological age” based on submaximal exercise performance with the application Myworkout GO. “Biological age” in the present study was defined as the average VO_2max_ for each sex and age in the general population [14]. The goal was to present both the general population and health care providers with a more accurate, easy to understand, and practical version of risk estimate. This might initially increase physical activity and improve exercise habits in the population. The aim of the present study was to evaluate the accuracy of predicting VO_2max_ from submaximal exercise using the application Myworkout GO. The hypothesis was that VO_2max_ predicted by the commercial smartphone application Myworkout GO would be significantly similar to direct VO_2max_ assessments.

## Methods

### Study Design and Participants

In this criterion-related validity design, study participants were recruited from universities, workplaces, athletic clubs, and senior organizations. Participants with previously diagnosed cardiovascular disease were excluded from this study. The intention was to recruit healthy people at different levels of CRF. Table 1 shows the main characteristics of the participants.

**Table 1:** Descriptive characteristics.

Characteristics		Total (n=162)		Men (n=104)			Women (n=58)			*P* value^a^
		Mean (SD)	Minimum-maximum	Mean (SD)	Minimum-maximum	Mean (SD)		Minimum-maximum		
Age (years)		38 (16)	17-73	30 (14)	17-71	50 (11)		30-73	<.001	
Body mass (kg)		79 (12)	51-128	81 (12)	60-128	76 (12)		51-102	.01	
Height (cm)		176 (8)	158-197	180 (7)	160-197	168 (4)		158-176	<.001	
**VO_2max_^b^ (mL/kg/min)**										
	Direct	49 (14)	19-79	57 (11)	31-79	36 (8)		19-54	<.001	
	Indirect^c^	50 (14)	17-77	57 (11)	30-77	36 (7)		17-53	<.001	

^a^Difference between men and women.

^b^VO_2max_: maximal oxygen consumption.

^c^VO_2max_ calculated by the application Myworkout GO.

### Ethics Approval

Review of the study design was undertaken by the Committee for Medical and Health Research Ethics in Norway who determined that a full committee review was not required given the healthy population. According to university policy, the study was submitted and approved by the institutional research board at the Norwegian University of Science and Technology and was performed in accordance with the Declaration of Helsinki (review number: NTNU/MH/ISB/JH/010919). All participants gave their written informed consent to participate after having reviewed oral and written information about the study and the procedures.

### Instruments

A calibrated motorized treadmill (TX200 GymSport, Trondheim, Norway) was used for both the VO_2max_ tests and Myworkout GO application assessements in this study. All measurements of pulmonary gas exchange were obtained using a Cortex Metamax II portable metabolic test system (Cortex, Leipzig, Germany). The participants used a face mask with a head cap assembly. The volume transducer for the Metamax system was connected to the face mask, together with a tube that collected samples of the gas concentration in the mask. This system was connected to a personal computer. The measurements were recorded every 10 seconds. The portable Metamax II metabolic test system offers an opportunity to measure all ventilatory parameters, VO_2_ and carbon dioxide output, and ambient air temperature and pressure. The ventilation volume transducer is a digital Triple-V turbine that measures a volume range of 0.0 L/s to 14.0 L/ s, with an accuracy of 1.5%. To analyze the oxygen concentration, a Zirconium sensor was used. The oxygen concentration range for the sensor is between 0 vol % and 25 vol %, with an accuracy of <0.1 vol %. Carbon dioxide was analyzed by an infrared sensor with a range from 0 vol % to 10 vol % and an accuracy of <0.1 vol %. Prior to the tests, the volume transducer was calibrated with a 3-L standardized calibration syringe (Hans Rudolph Jäger GmbH, Hoechberg, Germany). The gas concentration sensor was calibrated with ambient air and a chemically standardized calibration gas with 16% O_2_, 4% CO_2_, and 80% nitrogen (SensorMedics Corporation, Yorba Linda, CA).

Myworkout GO is an application accessible for both Android and iOS smartphones and gives timing information for performing a 4x4-minute workout. Myworkout GO has a specific algorithm for the prediction of VO_2max_ that will not be disclosed. The algorithm is based on completed amount of work (speed and inclination) during the 16-minute high aerobic intensity training that is manually registered in the app after completion of the HIIT session. Based upon the lineaer relationship between work and VO_2max_ [5], the application is able to evaluate the relative training intensity without wearing a HR monitor.

### Test Protocols

The study consisted of 3 experimental tests in randomized order on nonconsecutive days. The tests were performed within a maximum period of 2 weeks. One test was a direct VO_2max_ test on the treadmill, while Myworkout GO used a standardized HIIT protocol on the other 2 days. The highest predicted VO_2max_ value was used, blinded for directly measured results. Participants’ preparations consisted of not carrying out extreme exercise the day before the tests, not eating or drinking in the 2.5 hours before the tests, and not using tobacco in the 2 hours before the tests.

The VO_2max_ protocol on the treadmill involved a 10-minute warm-up period at about 70% of estimated maximum HR (HR_max_) based on the standard formula from the American College of Sports Medicine [15]. The test started after mounting the face mask and connecting it to the Metamax system. The workload was adjusted based on information about each participant’s weekly physical exercise level and treadmill practice. The participants typically started at the speed at which they finished their warm-up period. VO_2_ was measured constantly as the speed of the treadmill was increased every minute. This continued until the participant reached exhaustion after about 5 minutes to 8 minutes. To ensure that VO_2max_ was reached, the participants were encouraged to continue as long as possible so that a leveling off of VO_2_ occurred [1]. A plateau was displayed by all participants at the end of the test, confirming VO_2max_.

The HIIT protocol used in Myworkout GO was performed individually on the treadmill, walking or running, and consisted of a 6-minute warm-up at “talking speed.” Then, the participants underwent a 4x4-minute interval training (breathing heavy but with no obvious feeling of lactic acid accumulation), interrupted by 3 minutes of active rest periods at “talking speed” between each interval [1]. The 2 HIIT sessions were performed in a supervised setting by an exercise physiologist; however, the exercise itself was guided by the app, with the following instructions:

1. Walk or run uphill for the 6-minute warm-up at moderate intensity (talking pace).
2. Perform 4x4-minute intervals at an intensity at which you are breathing heavily after 2 minutes but do not feel any discomfort or stiff legs.
3. After the 4 minutes of high intensity, you should be able to do 1 more minute, and when you have completed the 4x4 minutes, given an active break, you should be able to do 1 more 4-minute interval.
4. Take 3-minute active breaks at talking pace between each interval.
5. Perform a 3-minute cooldown.

**Figure 1:**
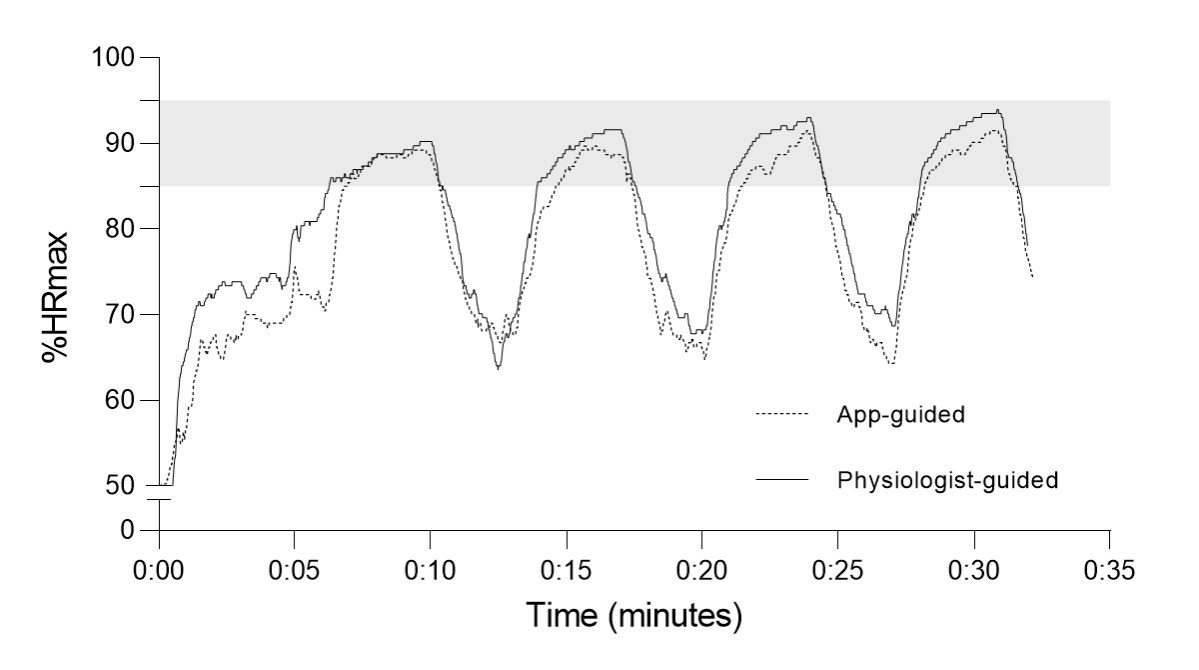
Examples of heart rate response to 4x4 high aerobic intensity interval training (HIIT) in healthy, young participants guided either by a physiologist or mobile application. The shaded area represents target intensity during the high-intensity intervals (85%-95% of maximum heart rate [HRmax]).

Since HR was not measured during the HIIT sessions, a randomized controlled pilot study was conducted prior to this study. The aim was to verify whether individuals can achieve the target intensity zone during HIIT when they either receive guidance by an exercise physiologist based on subjective feeling and observed level of exertion or simply follow the guidelines provided by the application Myworkout GO. For this purpose, 6 healthy, young individuals (4 men, 2 women; 20-30 years old) were recruited and randomized to a physiologist-guided (n=3) or an app-guided group (n=3). Every individual was advised to perform 3 HIIT sessions within 3 weeks on nonconsecutive days. HR was measured at the upper arm using a Polar OH1 monitor (Polar Electro Oy, Kempele, Finland). The Polar OH1 was recently validated with the gold standard for HR measurement, electrocardiography [16]. Both researchers and participants were blinded for HR during the pilot study. An example of the HR response for each group is presented in Figure 1. For statistical analysis, 4 data points per HIIT session were extracted, 1 average data point (in % of the individuals’ HR_max_) from the third minute of every interval.

### Statistical Analysis

Statistical analyses were performed using SPSS version 26 (IBM Corp, Armonk, NY). Means and standard deviations were computed for all the participants, and the measured variables are reported using descriptive statistics. Student *t* tests and linear regressions were used to calculate comparisons between the different means and variables in the tables and figures. Pearson correlation was performed to find the relationship between direct VO_2max_ and VO_2max_ estimated from Myworkout GO. Further, a Bland-Altman plot was used to describe the agreement of the 2 methods. In all statistical analyses, significance was accepted at *P*<.05. The figures were constructed using GraphPad Prism 8 (GraphPad Software, San Diego, CA).

## Results

**Figure 2:**
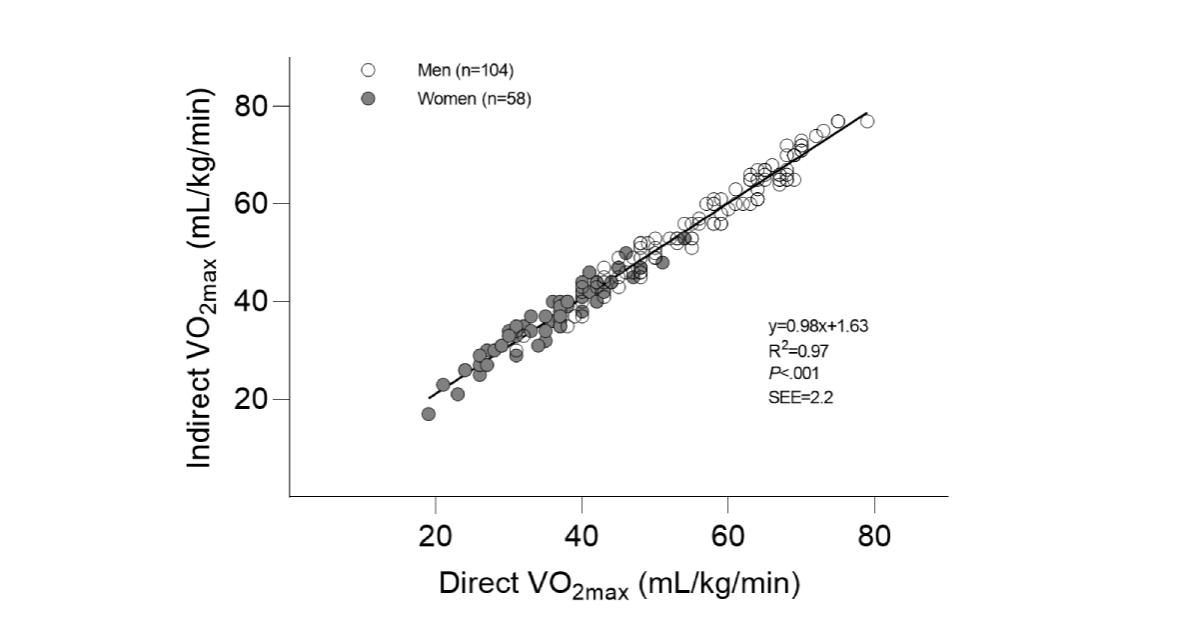
For all participants (n=162), the linear relationship between direct maximal oxygen consumption (VO_2max_) and predicted VO_2max_ calculated with the application Myworkout GO. SEE: standard error of the mean.

**Figure 3:**
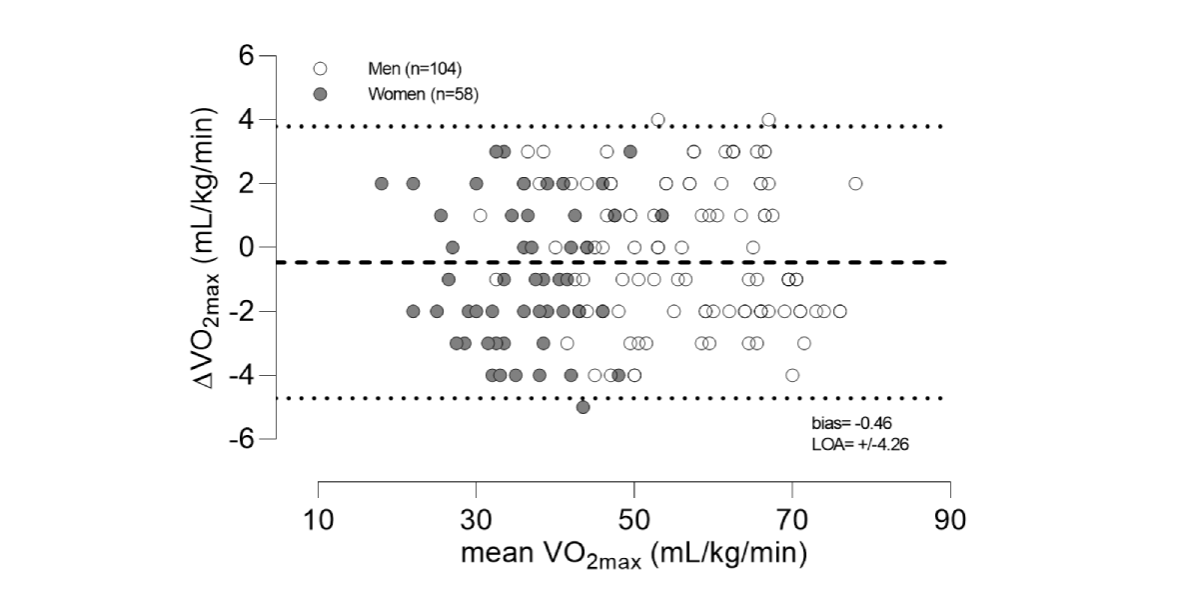
Bland-Altman plot showing the mean direct and predicted maximal oxygen consumption (VO_2max_) assessments plotted against the difference (Δ, direct - predicted) of the assessments (n=162). Bias is shown by the dashed line, and the 95% limits of agreement (LOA) are indicated by the dotted lines.

Participants included 162 healthy volunteers, 58 women and 104 men, between 17 years and 73 years of age. There were no significant differences between direct measurements of VO_2max_ and indirect calculations by Myworkout GO in all participants (Table 1) nor were there significant differences when the participants were divided into men and women. The direct and predicted VO_2max_ values were highly correlated, with an R^2^ of 0.97 (*P*<.001) and SEE of 2.2 mL/kg/min (4.5%; Figure 2), with no sex differences. The Bland-Alman plot for the direct and predicted VO_2max_ values is presented in Figure 3. The group of women were significantly older, had lower body mass and height, and had a significantly lower VO_2max_ than men (Table 1). Table 2 shows the age distribution among all the participamts.

**Figure 4:**
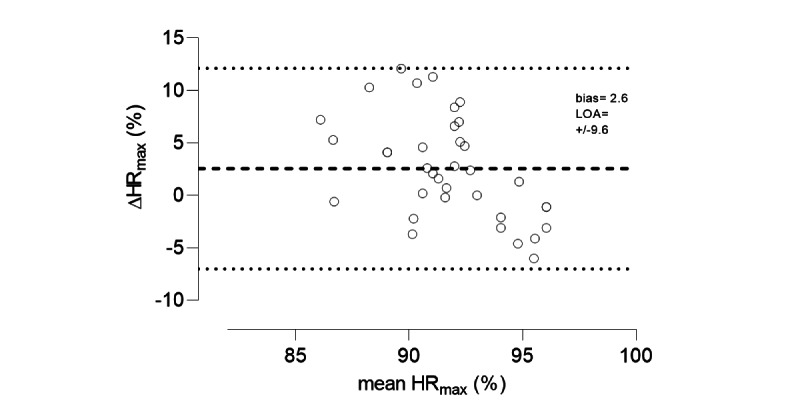
Bland-Altmann plot of mean heart rate (HR) response for physiologist-guided and app-guided groups (n=6) for all intervals plotted against the difference (Δ, physiologist-guided – app-guided) in HR between groups. Data are presented as percentage (%) of the individual’s maximum HR (HRmax). Bias is shown by the dashed line, and the 95% limits of agreement (LOA) are indicated by the dotted lines.

Results from the pilot study (n=6) revealed no significant difference between physiologist-guided and app-guided %HR_max_ in the first (mean 90.9, SD 2.4% vs mean 87.8, SD 3.8%; *P*=.05), second (mean 93.1, SD 2.6% vs mean 90.3, SD 4.2%; *P*=.11), third (mean 93.8, SD 2.1% vs mean 91.4, SD 4.5%; *P*=.18), and fourth (mean 94.4, SD 1.6% vs mean 92.3, SD 4.5%; *P*=.23) intervals. A typical example of the HR response for 1 participant in each group is presented in Figure 1. These findings were supported by the Bland-Altman plots, with all data points being within the 95% levels of agreement (Figure 4).

**Table 2:** Age distribution (n=162).

Age (years)	n
17-30	70
30-40	18
40-50	28
50-60	32
60-70	10
>70	4

## Discussion

### Principal Findings

The major novel finding of this study was no significant difference between direct VO_2max_ measurement (“gold standard”) and the predicted VO_2max_ measurement using the application Myworkout GO. The 2 methods were highly correlated (R^2^=0.97, *P*<.001), with an SEE of 2.2 mL/kg/min, which is equal to 4.5% of the average VO_2max_ in the total sample (mean 49, SD 14 mL/kg/min). The HIIT exercise in the app was tolerated well by the participants, and no adverse events were reported. Additionally, the pilot study demonstrated that the target intensity zone was reached. The calculated means and SEs for the physiologist-guided %HR_max_ (mean 93.0, SE 0.4%) and app-guided %HR_max_ (mean 90.5, SE 0.7%) exercise for all participants were not significantly different. Based on these results, we concluded that both methods guided individuals to the correct intensity zone (85%-95% HR_max_).

### Comparison With Prior Work

Compared with VO_2max_ reference data on a treadmill from 3816 healthy men and women aged 20 years to 90 years from the Norwegian population, our data were similar [17]. The baseline VO_2max_ of the male group (mean age 30, SD 13 years) was similar to the reference data in the age group of 20-30 years (mean 57, SD 10 mL/kg/min vs mean 54, SD 8 mL/kg/min) [17]. The female group (mean age 50, SD 13 years) was also similar to the reference data in the age group of 40-50 years (mean 35, SD 7 mL/kg/min vs mean 38, SD 8 mL/kg/min) [17]. In comparison, Edvardsen et al [14] presented normative VO_2max_ data from 759 male and female participants in Norway and reported lower numbers for both men in the age group of 20-30 years (mean 49, SD 10 mL/kg/min) and women in the age group of 40-50 years (mean 33, SD 6 mL/kg/min).

More recently, the Fitness Registry and the Importance of Exercise National Database published VO_2max_ reference standards for 4611 adult men and 3172 women (20-79 years old) obtained from direct VO_2max_ measurements [18]. Compared with the results from Edvardsen et al [14], these average numbers from the US population are similar for men (mean 48, SD 11 mL/kg/min) but slightly lower for women (mean 28, SD 8 mL/kg/min).

The exercise testing modality has a significant impact on results; the values were 10% to 20% lower when using a cycle ergometer compared with a treadmill in untrained individuals [5]. Moreover, study population, test protocol, exclusion criteria prior to testing, and type of equipment used are some reasons why differences occur across studies. Physical activity level and a smaller sample size may well explain differences in VO_2max_, both between the reference data and this study.

### Physical Activity, CRF, and Health

Physical activity can act as primary prevention against more than 35 chronic diseases and should thus be prescribed as medicine [19]. There is, however, a need to translate basic research to clinical practice to make more people move. It is crucial to note that “Nonexercise estimated CRF should not be viewed as a replacement for objective assessment of CRF, especially in some at-risk patient populations” [4]. This is illustrated by the SEE for their equations ranging from an SEE of 3.0 mL/kg^/^min (9.7%; R^2^=0.74) reported by Cao et al [20] to an SEE of 5.7 mL/kg^/^min (12.8 %; R^2^=0.61) reported by Nes et al [21]. Ross and collaborators [4] also concluded that CRF should be measured in clinical practice since it can provide additional information that influences patient management.

After adjustment for age and other risk factors, CRF has been documented to be a strong independent marker of risk for cardiovascular and all-cause mortality. A meta-analysis by Kodama et al [22] extracted 33 studies including nearly 103,000 participants. For every metabolic equivalent (resting metabolic rate or oxygen consumption of 3.5 mL/kg/min) increase in CRF, 13% and 15% reductions in cardiovascular and all-cause mortality, respectively, were observed.

Harb and colleagues [9] calculated the risk of death in their study of 126,356 participants (1991-2015), adjusted for sex, cardiovascular disease, type 2 diabetes, statin use, hypertension, smoking, and body mass index. They concluded that “biological age” based on CRF better predicts all-cause mortality compared with chronological age. Every effort should be undertaken to improve CRF in sedentary adults, since half the reduction in all-cause mortality occurs between the least-fit group and the next least-fit group. However, higher CRF is associated with reduced risk even among participants within the low-fit [23] or low-risk group [24].

CRF is often neglected as a risk marker compared with conditions treatable with drugs or invasive procedures [18]. Wearable technologies claim to provide accurate measurements of HR, energy expenditure, and VO_2max_. However, Wallen et al [25] demonstrated that all tested devices measuring HR via photoplethysmography underestimated HR and especially energy expenditure. Thus, it would limit their use for evaluating CRF and training intensity and acting as a weight loss aid. Bent et al [11] documented that wearable optical HR sensors had, on average, an absolute error during activity 30% higher than during rest. Digital biomarker interpretation must take the data quality into account when making health-related decisions.

### Clinical Perspectives

Considering the strong independent value of CRF as a risk marker for cardiovascular and all-cause mortality [22], evaluation of CRF is of utmost importance in a vast number of clinical populations. Patients may encounter different central or peripheral pathologies that cause limitations set by metabolic demands or by one or more of the components of the integrated O_2_ transport pathway [26], limitations that may inhibit these individuals’ maximal exercise capacity and ability to reach a plateau of VO_2_, consequently attaining VO_2peak_ instead of VO_2max_. Whether Myworkout GO’s algorithm will be able to predict symptom-limited VO_2peak_ as it relates to different patient populations, with similar accuracy as presented in this study with healthy participants, is yet to be determined. However, the submaximal HIIT exercise utilized by the application has high clinical value, as it indeed represents the current state of symptom-limiting exercise capacity. It presents a unique evaluation of exercise tolerance while under controlled conditions and assesses the response from all elements involved in the O_2_ pathway, from the atmosphere to the working mitochondria. These results may provide valuable information for clinical practice, both diagnostically and in terms of exercise treatment.

### Practical Applications and Future Directions

Cars, elevators, remote controls, and other modern devices all help to engineer physical activity out of people’s lives. Engineering physical activity back into their lives and informing them of the health benefits are paramount. It has also been documented that people will miss less work and be more productive [27]. We sought to close the gap between knowledge and practice. It is well established that exercise is medicine and utilizing smartphone applications, such as Myworkout GO, creates an accessible solution to administer exercise worldwide. The application provides an opportunity to revolutionize health care, particularly in communities with traditionally limited health care access. Consequently, investigations targeting the accuracy of exercise-based CRF prediction in patient populations are warranted. Outside the clinical setting, smartphone applications can in fact utilize available technology such as GPS, barometric pressure, and high-quality map data to automatically track and generate the required information from a free living situation to predict CRF from outdoor workouts. This opens up the possibilities for future research and, more importantly, the population to health-enhancing activity while simultaneously receiving evaluation of relevant health information.

### Strengths and Limitations

There are both strength and limitations to this study. One limitation is the possibility that people who volunteer for participation in a exercise research study are experienced with physical exercise and subsequently have high internal motivation to adhere to the research protocol, causing a selection bias. However, comparison of CRF with reference data [17] revealed that the results for both men and women in this study where similar to those of the general Norwegian population, indicating comparable populations.

The controlled laboratory setting utilized in this study is a strength, as this type of investigation gives great insight into the genuine accuracy of the algorithm when there is compliance with the protocol. However, caution must be taken as to not indiscriminately extrapolate the results from this study to a free-living situation where sincere adherence to the protocol may be muddled with the intention to comply. Correct execution of both the HIIT exercise and in-app registration is crucial for CRF prediction accuracy. Consideration of not only human error but also potential technical complications such as uncalibrated exercise equipment as factors influencing the accuracy of the CRF prediction must occur. Ultimately, the algorithm simply works with what it is given.

Although outside the scope of this study, low-threshold, easily available, outdoor exercise is appealing for many. Speed and inclination from outdoor walking or running can be attained and automatically registered by Myworkout GO and utilized to predict CRF. However, it is prudent to remember that potential limitations to such measurements may exist. For instance, GPS data accuracy and type of surface will influence the input to the CRF prediction, even though the exercise effect of the HIIT sessions may be similar. Thus, to increase the extrapolatory value to free-living situations, compliance with the HIIT guidance and standardization of the test setting should be emphasized.

### Conclusion

There was no significant difference between direct VO_2max_ measurement and predicted VO_2max_ measurement using the application Myworkout GO in the total sample. The 2 methods were highly correlated, with an SEE of 2.2 mL/kg^/^min, which is equal to 4.5% of the average VO_2max,_ in healthy participants who comply with the protocol. The HIIT session (4x4 minutes) incorporated in the application Myworkout GO was tolerated well by the participants. Another goal with Myworkout GO is to give the most time-efficient recommendations to improve CRF for both the healthy population and patients. Precise and effective digital health applications have the potential to transform health care through inexpensive and convenient monitoring outside the clinic.

## Abbreviations

CRF: cardiorespiratory fitness
HIIT: high aerobic intensity interval training
HR: heart rate
HRmax: maximum HR
SEE: standard error of the estimate
VO_2max_: maximal oxygen consumption

## Data Availability

The data that support the findings of this study are available from the corresponding author upon reasonable request.
